# Evaluating the metropolitan public health preparedness for pandemics using entropy-TOPSIS-IF

**DOI:** 10.3389/fpubh.2024.1339611

**Published:** 2024-03-07

**Authors:** Jin Liu, Allen Wood Liu, Xingye Li, Hui Li, Wenwei Luo, Wei Chen

**Affiliations:** ^1^College of Information Engineering, Shanghai Maritime University, Shanghai, China; ^2^Shanghai Experimental School International Division, Shanghai, China; ^3^Faculty of Education and Human Development, The Education University of Hong Kong, Hong Kong, Special Administrative Region, Tai Po, China; ^4^College of Early Childhood Education, Shanghai Normal University, Shanghai, China; ^5^College of International Education, Shanghai Business School, Shanghai, China

**Keywords:** TOPSIS, entropy-TOPSIS-IF, fuzzy theory, multicriteria decision making, public health preparedness, preparedness on pandemics

## Abstract

**Introduction:**

Metropolitan governance’s efficacy is regularly gauged by its capability for public health preparedness, a critical component, particularly in the post-pandemic climate, as global cities reassess their mitigation abilities. This process has broader implications, curbing mortality rates and amplifying sustainability. Current methodologies for preparedness assessment lean primarily on either Subjective Evaluation-Based Assessment (SBA), predicated on experts’ input on various capacity indicators, or they opt for Data-Based quantitative Assessments (DBA), chiefly utilizing public statistic data.

**Methods:**

The manuscript discusses an urgent need for integrating both SBA and DBA to adequately measure Metropolitan Public Health Pandemics Preparedness (MPHPP), thus proposing a novel entropy-TOPSIS-IF model for comprehensive evaluation of MPHPP. Within this proposed model, experts’ subjective communication is transformed into quantitative data via the aggregation of fuzzy decisions, while objective data is collected from public statistics sites. Shannon’s entropy and TOPSIS methods are enacted on these data sets to ascertain the optimal performer after normalization and data isotropy.

**Results and discussion:**

The core contribution of the entropy-TOPSIS-IF model lies in its assessment flexibility, making it universally applicable across various contexts, regardless of the availability of expert decisions or quantitative data. To illustrate the efficacy of the entropy-TOPSIS-IF model, a numerical application is presented, examining three Chinese metropolises through chosen criteria according to the evaluations of three experts. A sensitivity analysis is provided to further affirm the stability and robustness of the suggested MPHPP evaluation model.

## 1 Introduction

Since the emergence of Severe Acute Respiratory Syndrome in 2003, recurring threats to global public health and sustainable development due to sporadic outbreaks of widespread infectious diseases have increasingly become a reality. Evidence from previous studies (1) supports the notion that the prevalence of emerging infectious illnesses has escalated over the years, a trend attributed to numerous factors such as increased international travel and commerce, burgeoning population density, evolving human-wildlife interaction dynamics, and even ecological shifts due to global warming. These difficulties underscore the importance of systematically evaluating and enhancing Metropolitan Public Health Pandemics Preparedness (MPHPP) as a pressing priority for nations worldwide.

The traditional methodologies applied in the assessment of MPHPP predominantly rely on subjective evaluation-based assessment (SBA), such as the RUHSA (Rapid Urban Health Security Assessment) tool (2) or data-based assessment (DBA) using second-hand public data (3). While SBA methodologies provide vital perspectives on urban health security system weaknesses and assist authorities in formulating strategic remedies, they often fail to provide an effective reference for urban governance due to a lack of objective data analysis (4). On the other hand, DBA, as proven effective by studies like that of Chu et al. (5), contributes valuable insights into the significant influence of urban government’s role on MPHPP during pandemics. Yet, their reliance on historical data may lead to overlooking non-quantifiable factors that SBA methods consider.

In light of these limitations, this paper introduces an innovative approach: entropy-TOPSIS-IF amalgamating the strengths of both SBA and DBA methodologies to provide a rigorous and comprehensive MPHPP evaluation framework. Specifically, subjective linguistic evaluation results were converted to numerical data by the fuzzy set theory approach. Objective data achieved from the official statistics website is combined with converted numerical data to evaluate MPHPP comprehensively. Accordingly, the integration of SBA and DBA can effectively address the constraints seen in prior research and enhance the effectiveness of the MPHPP evaluation. The following questions guided this study:

RQ1: How MPHPP could be evaluated through a combination of subjective judgment and objective data?
RQ2: Is the entropy-TOPSIS-IF model suitable and sensitive enough for measuring the MPHPP?

## 2 Literature review

Research on MPHPP assessment methodologies is crucial for enhancing the capacity of a metropolis to respond to the next pandemic and ensuring the population’s health. Studies in the literature can be categorized as SBA and DBA.

### 2.1 Subjective evaluation-based assessment

During the early exploration of the SBA method, most researchers concentrated on determining which factors should serve as key indicators for evaluating MPHPP. Sharifi (6) conducted an analysis of 36 specific tools that were chosen to assess MPHPP. A comprehensive analytical framework was devised to provide six distinct criteria for evaluating the performance of these tools. Nevertheless, the assessment result has shown that there has been only modest success in meeting these requirements. Additionally, the environmental factor has been given comparatively less emphasis regarding comprehensiveness. Mahmood et al. (7) conducted a qualitative study on the key factors leading to the low MPHPP. Eventually, “Mismanagement” and “Inefficient use of resources” were the most significant challenges to urban health financing during the pandemic. In addition, “Insufficient information sharing,” “Uneven distribution of health personnel,” and “Severe shortage of human resources” were also considered as the key factors resulting in low MPHPP of Dhaka city. Zhou et al. (8) performed a qualitative study using in-depth interviews to examine the critical indicators for enhancing the MPHPP in Guangzhou, China. The results show that “close collaboration between the government and the community,” “strict control of population movement,” and “massive deployment of nucleic acid testing points and medical facilities” are the keys to Guangzhou’s success in facing the sudden epidemic. Moussallem et al. (9) conducted an evaluation of MPHPP in Lebanon in the form of a qualitative study based on semi-structured interviews. The results show that “health financing,” “medical human resources,” and “government governance capabilities” are critical for areas with severe political and economic pressure.

While these studies successfully identified key factors affecting MPHPP, they did not establish a readily available indicator system or assessment tool specifically designed for evaluating MPHPP, thus lacking feasibility for practical application. To address this limitation, George et al. (10) put forward the Regional Epidemic Preparedness Index, which includes four key indicators: schedulable resources, information dissemination capacity, disaster management plan, and number of active NGOs. Shi et al. (11) utilized the crip-set qualitative comparative analysis approach to investigate the influencing variables that affect urban resilience during the pandemic outbreak and selected 7 key factors, including “population density,” “economic conditions,” “public facilities,” etc.

Although these methods can provide some practical guidance as assessment tools for MPHPP to some extent, they ignore the relative significance of each evaluation factor in MPHPP assessment. Therefore, to effectively quantify the significance of each indicator, some researchers have considered introducing fuzzy TOPSIS-based methods (12) for evaluation. Cheng et al. (13) proposed an MPHPP evaluation method based on a fuzzy comprehensive evaluation approach. Specifically, an evaluation index system for MPHPP was established, including a total of 8 indicators. Then, the fuzzy logic algorithm is used to quantify these indicators, while the weight of each indicator is determined by the utilization of analytic hierarchy process. Rezaei et al. (14) proposed a multicriteria MPHPP assessment method. They screened out 20 criteria for measuring MPHPP through expert questionnaires and analyzed the vulnerability of 19 urban areas in Qazvin by means of the analytic hierarchy process. Yi et al. (15) evaluated MPHPP during the pandemic in 16 countries using fuzzy set qualitative comparative analysis. The results show that the “Medical capacity” holds the most significance in measuring MPHPP.

The introduction of methods like fuzzy assessment has greatly advanced the research on the SBA method. However, the subjective selection of influencing factors still lacks sufficient persuasiveness and generalizability across different metropolitan scenarios.

### 2.2 Data-based assessment

Considering the lack of objectivity of the SBA method, some researchers have tried to evaluate MPHPP by analyzing historical data that may be related to MPHPP. Prieto et al. (16) proposed an Urban Vulnerability Assessment method, UVA, which assesses MPHPP by calculating the Urban Vulnerability Index, composed of multiple vulnerability factors. The UVA can be applied to different audiences and different scales for prior assessment and decision-making of MPHPP. Effat et al. (17) proposed a Spatial Multicriteria Evaluation model to assess the PHTP of Assiut City, which evaluates 13 indicators related to housing, socio-environmental, and economic conditions using standardized, weighted, and aggregated methods. The result revealed that vulnerable areas tend to have the highest proportion of slums, highest population density, highest urban growth rates, and poor service connectivity, which also implies that “economic level,” “population density,” and “service connectivity” may be the critical factors in assessing MPHPP. Li et al. (18) proposed an urban epidemic hazard metric to assess MPHPP in Chinese prefectural districts. The urban epidemic hazard metric, built through epidemic simulations of multi-layered transportation networks, offers a quantitative elucidation of the occurrence wherein small-to-middle scale cities in China emerged as COVID-19 hotspots during the initial wave in 2021. The results of the analysis show that local population density and intercity transportation construction may be the main factors affecting MPHPP. Chen et al. (19) conducted a quantitative study on the key influencing factors of MPHPP using multiple linear regression. The results show that population density, healthcare infrastructure, and size of urban economic activity are positively correlated with the inflow risk of a pandemic. Among them, the scale of economic activities is the main factor in the rise of the pressure of urban pandemic inflows and the burden on medical resources. Wang et al. (20) analyzed the Metropolitan Region of Amsterdam via established social and environmental urban vulnerability frameworks. Their research aimed to evaluate the implications and responses to pandemics, explicitly focusing on assessing MPHPP. To do this, the researchers identified suitable indicators related to spatial, environmental, and socio-demographic factors. Lin et al. (21) established an urban Pandemic Vulnerability Index for measuring the MPHPP. The construction of the Pandemic Vulnerability Index involves utilizing 140 statistical data and 10 dynamic variables. Furthermore, to aggregate components and forecast vulnerability, the LambdaMART algorithm is employed. Zhou et al. (22) employed the TOPSIS entropy weight technique and a coupled coordination model to evaluate the MPHPP of six metropolitan regions situated along the Sichuan-Tibet Railway in China during a five-year period from 2015 to 2020. The evaluation encompassed several dimensions: economic, social, environmental, and infrastructure. In addition, correlation and gray correlation analyses are employed to ascertain the key determinants that influence the MPHPP.

In addition, in recent years, many studies (23, 24) have indirectly conducted a comprehensive assessment of MPHPP by evaluating “urban resilience,” which refers to an urban’s ability to withstand and recover from the impacts of an epidemic. Chen et al. (25) proposed an Urban Resilience Evaluation Index (UREI) via the spatiotemporal analysis of urban in the Yangtze River Delta region of China, which includes four dimensions: (i) Economy, (ii) Ecology, (iii) Infrastructure, and (iv) Social system. Suleimany et al. (26) developed a comprehensive framework to assess urban pandemic resilience, which includes five dimensions: (i) Institutional, (ii) Social, (iii) Economic, (iv) Infrastructure, and (v) Demographic. Zhang et al. (27) introduced a comprehensive urban resilience framework that is both dynamic and systematic. This framework encompasses four key indicators: governance, infrastructure, socio-economy, and energy-material fluxes. Their Nanjing and Wuhan case studies demonstrate that strict control policies may lead to a significant decline in urban resilience. Yuan et al. (28) extracted the citizen complaint data of each urban during the COVID-19 pandemic with text mining technology and quantitatively analyzed its temporal changes. The results show that the economic system and housing construction system, urban welfare system, environmental system, travel system, and transportation system are the factors that receive the most complaints.

The rest of the article is organized as follows. In the method section, basic concepts of the fuzzy set theory, the analysis model, entropy-TOPSIS-IF, and evaluation indicator determination process were presented. In section 4, we presented a case study evaluating MPHPP in three metropolises using the entropy-TOPSIS-IF model. Section 5 presented our reflection on the advantages and disadvantages of this model and how the findings of our case study bring new insights into MPHPP evaluation and policy recommendations. Limitations and future work were also presented in this section. In section 6, the conclusion of the article was presented.

## 3 Methods

A novel multicriteria decision analysis model, Entropy-TOPSIS-IF, was proposed to evaluate the MPHPP. Entropy-TOPSIS-IF integrated subjective evaluation and existing data secondary analysis. Specifically, subjective linguistic evaluation results were converted to numerical data by the fuzzy set theory approach. Objective data achieved from the official statistics website is combined with converted numerical data to evaluate MPHPP comprehensively.

Since the evaluation model of this study consists of two different sections and the subjects and methods involved in each section are different, the participants and the measures employed will be reported separately in each section. Before we recruited participants, this study was reviewed and approved by the Ethics Committee of Shanghai Normal University (No. 2023044).

### 3.1 Basic concepts of the fuzzy set theory approach

The conception of fuzzy set theory, also known as fuzzy logic, was employed to compute the current study (29). This innovative framework deviated from the conventional binary logic, which operates on the dichotomous “true or false” paradigm (i.e., the Boolean logic of 1 or 0), by introducing the concept of “degrees of truth” for characterizing the properties and behaviors of entities or systems. Many improved fuzzy methods have been developed for application in a variety of disciplines, including optimization of multi-objective problems and multicriteria decision-making (30, 31). We present two interconnected definitions of fuzzy set theory as follows.

### 3.2 Definition 1

A fuzzy set $\tilde{\alpha }$ within the universe of discourse X is characterized by its membership function $\mu_{\tilde{\alpha }}\left(x\right)$ , which assigns a real number between 0 and 1 to each element x in X. According to Kaufmann and Gupta (32), the grade of membership of x in $\tilde{\alpha }$ is denoted as $\mu_{\tilde{\alpha }}\left(x\right)$ . As the value of $\mu_{\tilde{\alpha }}\left(x\right)$ approaches unity, the grade of membership of x in $\tilde{\alpha }$ increases.

### 3.3 Definition 2

**Figure 1:**
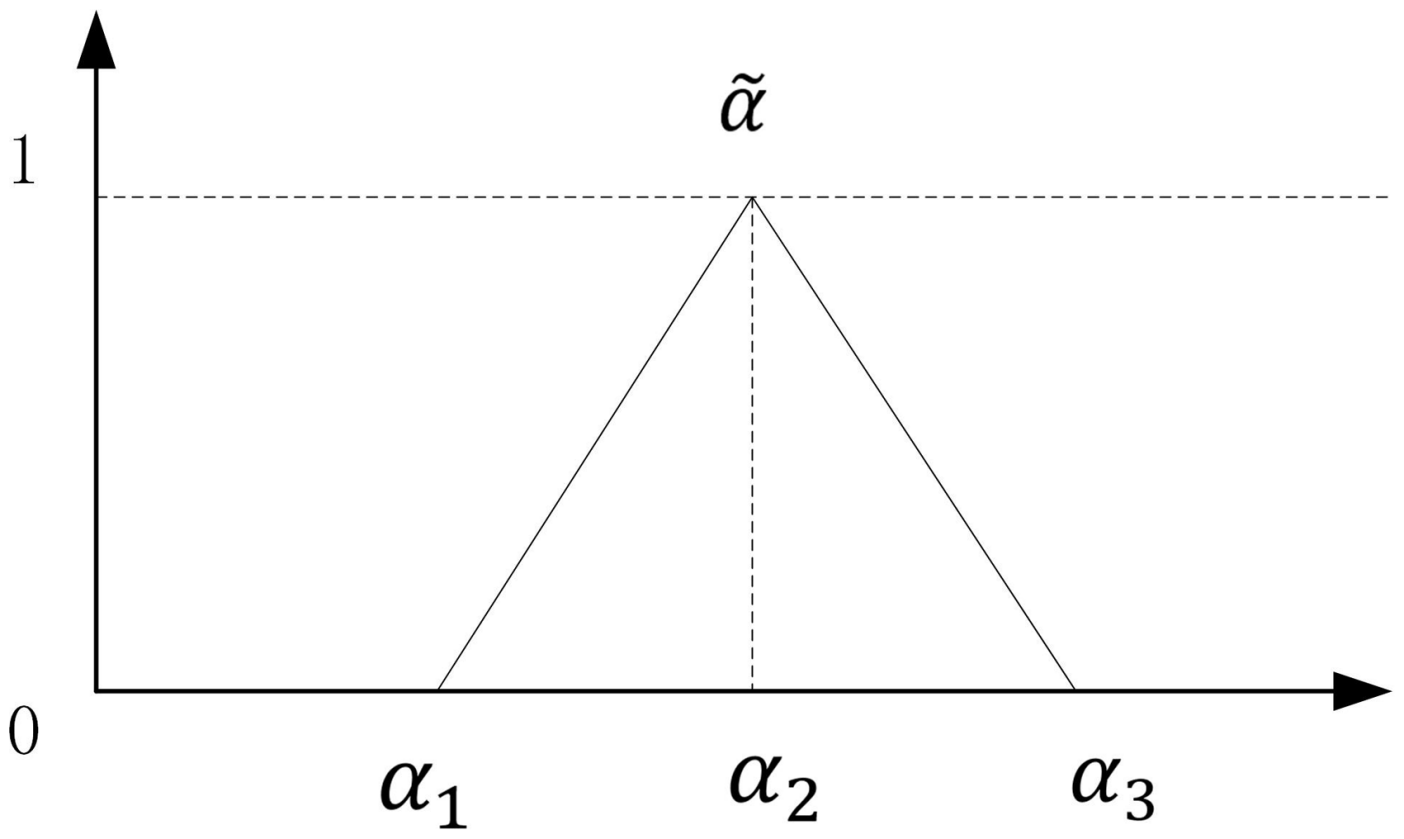
Triangular fuzzy number $\tilde{\alpha }$ .

A triangular fuzzy number can be denoted as a triplet, represented as $\tilde{\alpha }=\left(\alpha_{1},\alpha_{2},\alpha_{3}\right)$ . Figure 1 illustrates a triangular fuzzy integer denoted as $\tilde{\alpha }$ . The utilization of triangular fuzzy numbers in practical applications is widespread owing to their inherent simplicity in terms of both conceptual understanding and computational implementation (33–35). The triangular fuzzy number $\tilde{\alpha }$ is characterized by the membership function $\mu_{\tilde{\alpha }}\left(x\right)$ , which is expressed as Eq. 1


(1) $$\mu_{\tilde{\alpha }}\left(x\right)=\{\begin{array}{c} 0,x\leq \alpha_{1,}\\ \frac{x-\alpha_{1}}{\alpha_{2}-\alpha_{1}},\alpha_{1}\leq x\leq \alpha_{2}\\ \begin{array}{c} \frac{\alpha_{3}-x}{\alpha_{3}-\alpha_{2}},\alpha_{2}\leq x\leq \alpha_{3}\\ 0,x>\alpha_{3} \end{array} \end{array}$$

Let $\alpha_{1}$ , $\alpha_{2}$ , and $\alpha_{3}$ be real integers such that $\alpha_{1}$ < $\alpha_{2}$ < $\alpha_{3}$ . The maximum grade of $\mu_{\tilde{\alpha }}\left(x\right)$ , denoted as $\mu_{\tilde{\alpha }}\left(x\right)$ = 1, is obtained when evaluating the value of x at $\alpha_{2}$ . This result represents the most probable outcome of the evaluation data. The value of x at $\alpha_{1}$ corresponds to the minimum grade of $\mu_{\tilde{\alpha }}\left(x\right)$ , specifically $\mu_{\tilde{\alpha }}\left(x\right)$ = 0. This value represents the least likely outcome of the evaluation data. The variables $\alpha_{1}$ and $\alpha_{3}$ represent the minimum and maximum limits of the available area for the evaluation data. The constants indicate the inherent imprecision in the assessment data, as stated by Liang (36). The degree of fuzziness in the evaluation data decreases as the interval [ $\alpha_{1}$ , $\alpha_{3}$ ] narrows.

### 3.4 Linguistic variables and fuzzy rating

The utilization of conversion scales is a fundamental aspect of the fuzzy set theory, as it transforms linguistic variables into fuzzy numbers. A 1–9 scale will be adopted to evaluate targets in this article. Table 1 presents the subjective linguistic terms and fuzzy ratings employed to evaluate the objectives.

**Table 1:** Linguistic variables and fuzzy ratings for targets evaluation.

Linguistic variables	Fuzzy numbers
Very weak (VW)	(1,1,3)
Weak (W)	(1,3,5)
Qualified (Q)	(3,5,7)
Well qualified (WQ)	(5,7,9)
Very well qualified (VWQ)	(7,9,9)

### 3.5 The entropy-TOPSIS-IF model

**Figure 2:**
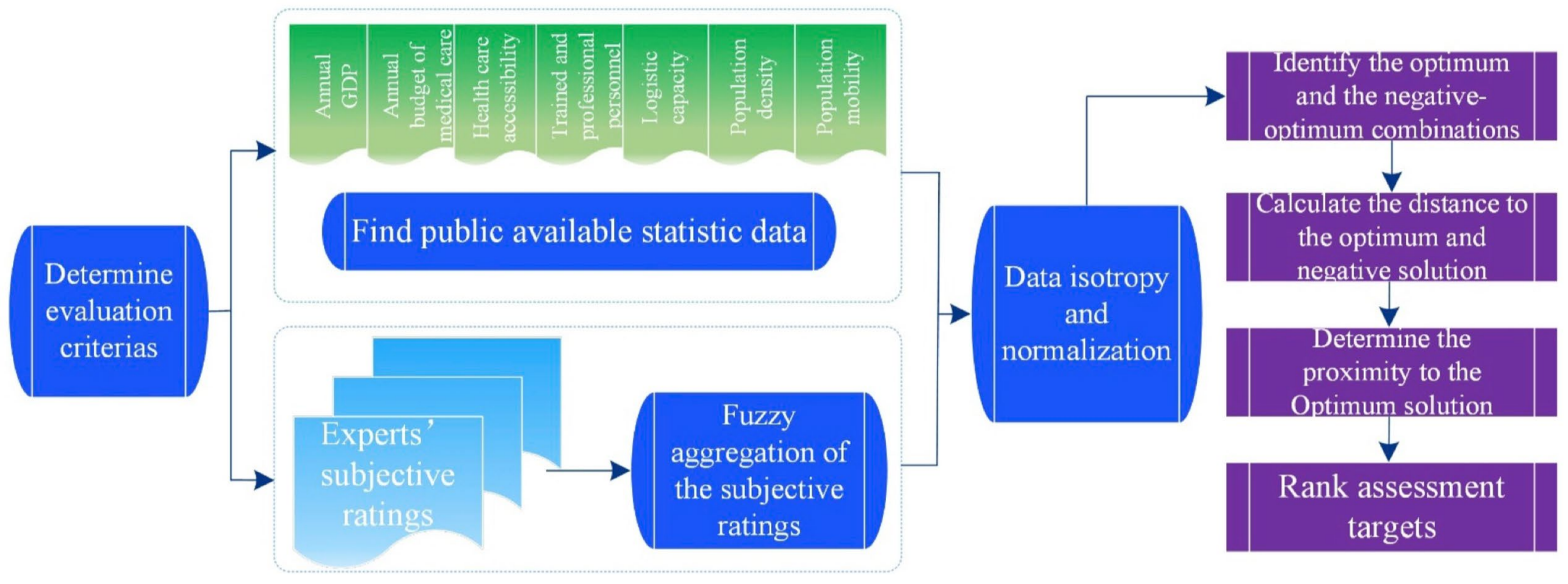
Flow of the proposed entropy-TOPSIS-IF.

Since entropy-TOPSIS-IF integrally analyze both subjective evaluations of linguistics ratings and object data obtained from publicly available data sources, these two distinct data types will be first obtained. After converting the subjective linguistic words to fuzzy triangular numbers, TOPSIS is used to combine all the criteria ratings and objective data to generate an overall score for each target. The overall flow of the entropy-TOPSIS-IF is shown in Figure 2.

**Step 1**: Ratings the target on each required subjective criterion.

Let us consider a scenario where there exists a set of J assessment targets denoted as $T=\left\{T_{1},T_{2},\cdots ,T_{j}\right\}$ . These targets are to be evaluated based on a set of n criteria denoted as $C=\left\{C_{1},C_{2},\cdots ,C_{n}\right\}$ . The weights assigned for each criterion are represented by $w_{i}=\left(i=1,\;2,\cdots ,\;m\right)$ . The rating given by each expert ${\tilde{R}}_{k}\left(k=1,\;2,\;\cdots ,\;k\right)$ for each assessment target $T_{j}\left(j=1,\;2,\;\cdots ,\;n\right)$ regarding the criteria $C_{i}\left(i=1,\;2,\;\cdots ,\;m\right)$ can be detonated as Eq. 2


(2) $$\begin{array}{l} \;C_{1}\;\;\cdots \;\;C_{2}\;\;\cdots \;C_{n}\\ {\tilde{R}}_{k}=\begin{array}{c} T_{1}\\ \vdots \\ \begin{array}{c} T_{2}\\ \begin{array}{c} \vdots \\ T_{m} \end{array} \end{array} \end{array}\left[\begin{array}{cc} \begin{array}{c} {\tilde{x}}_{11}\\ \vdots \end{array} & \begin{array}{c} \cdots \end{array}\;\begin{array}{c} {\tilde{x}}_{12}\\ \vdots \end{array}\;\begin{array}{c} \begin{array}{cc} \cdots & {\tilde{x}}_{1n} \end{array}\\ \begin{array}{cc} & \vdots \end{array} \end{array}\\ \begin{array}{c} {\tilde{x}}_{21}\\ \vdots \\ {\tilde{x}}_{m1} \end{array} & \begin{array}{ccc} & \begin{array}{c} {\tilde{x}}_{22}\\ \vdots \\ {\tilde{x}}_{m2} \end{array} & \begin{array}{c} \begin{array}{cc} & {\tilde{x}}_{2n} \end{array}\\ \begin{array}{cc} & \vdots \end{array}\\ \begin{array}{cc} & {\tilde{x}}_{mn} \end{array} \end{array} \end{array} \end{array}\right]\\ \left(i\;=\;1,\;2,\;\cdots ,\;m;j=1,2,\cdots ,n;\;k=1,2,\cdots K\right) \end{array}$$

with membership function $\mu_{{\tilde{R\;}}_{k}}\left(x\right)$

**Step 2.1**: The fuzzy ratings for the subjective criterion are aggregated.

If the ratings of experts are represented as triangular fuzzy numbers ${\tilde{R}}_{k}=\left(\alpha_{k},\;\beta_{k},\;\gamma_{k}\right)$ , where k=1,2,⋯,K , then the aggregated fuzzy rating can be expressed as Eq. 3


(3) $$\tilde{R}=\left(\alpha ,\;\beta ,\;\gamma \right)\text{,}k=1,2,\cdots ,K$$

where *α, β, γ* can be expressed as Eq. 4


(4) $$\alpha =\min_{k}\left\{\alpha_{k}\right\},\;\beta =\frac{1}{K}\overset{K}{\underset{k=1}{\sum }}\beta_{k},\;\gamma =\max_{k}\left\{\gamma_{k}\right\}$$

Then fuzzy rating and weight of the kth expert can be expressed as ${\tilde{x}}_{ijk}=\left(\alpha_{ijk},\;\beta_{ijk}\gamma c_{ijk}\right)$ and ${\tilde{w}}_{ijk}=\left(w_{jk1},\;w_{jk2},\;w_{jk3}\right)$ , respectively, where i represents the expert index (1, m), and j represents the criterion index (1, n). The calculation of the aggregated fuzzy ratings $\left({\tilde{x}}_{ij}\right)$ for targets in relation to each criterion can expressed as ${\tilde{x}}_{ij}=\left(\alpha_{ij},\;\beta_{ij},\;\gamma_{ij}\right)$ where they can be detonated as Eq. 5


(5) $$\alpha_{ij}=\min_{k}\left\{\alpha_{ijk}\right\},\;\beta_{ij}=\frac{1}{K}\overset{K}{\underset{k=1}{\sum }}\beta_{ijk},\;\gamma_{ij}=\max_{k}\left\{\gamma_{ijk}\right\}$$

The aggregated fuzzy weights $\left({\tilde{w}}_{ij}\right)$ for each criterion are then obtained as where they can be calculated as ${\tilde{w}}_{j}=\left(w_{j1},w_{j2},w_{j3}\right)$ Eq. 6


(6) $$w_{j1}=\min_{k}\left\{w_{jk1}\right\},w_{i2}=\frac{1}{K}\overset{K}{\underset{k=1}{\sum }}w_{jk2},w_{j3}=\max_{k}\left\{w_{jk3}\right\}$$

**Step 2.2**: Obtain objective data from public available statistic websites.

Objective data is obtained from the government’s official statistics websites. Assume that there are u criteria for the objective data used for DBA, which is detonated as $O=\left\{O_{1},O_{2},\cdots ,O_{u}\right\}$ , data obtained for each criterion can be expressed as $Y=\left\{y_{1},\;y_{2},\cdots ,\;y_{u}\right\}$ , then the objective data to be used in entropy-TOPSIS-IF can be expressed as $O=\left\{O_{1}=y_{1},\;O_{2}=y_{2},\;\cdots ,\;O_{u}=y_{u}\right\}$ .

**Step 3**: Data isotropy and normalizations

Data isotropy is performed using the equation $x^{\prime }=\frac{1}{x}\;\left(x>0\right)$ for every miniaturized index of the criteria. After that, data normalization is done by the following formula Eq. 7


(7) $$p_{ij}=\frac{x_{ij}}{\sum_{i=1}^{n}x_{ij}}$$

where x_ij_ is the numerical score for j_th_ criterion of i_th_ target.

**Step 4**: Get weight for each criterion by Shannon Entropy.

At this step, the data normalization process involves ensuring data isotropy, whereby all data points are transformed to a consistent assessment scale. The concept of information entropy is formally defined as Eq. 8


(8) $$E_{j}=-\frac{1}{\ln m}\overset{n}{\underset{j=1}{\sum }}p_{ij}\ln p_{ij}$$

and the weight is then calculated by Eq. 9


(9) $$w_{ij}=\frac{\left(1-E_{j}\right)}{\sum_{j=1}^{n}\left(1-E_{j}\right)}$$

**Step 5**: Identify the optimum and the negative- optimum combinations.

Following indicator politicization, we identify the optimum and negative-optimum solutions. The ideal combination has the optimal values for each criterion, whether maximized or minimized, while the negative-optimum one has the worst values. These reference points guide the subsequent evaluation, helping assess targets about these optimum states.

**Step 6**: Determine the distance to the optimum solution ( $\delta_{+}$ ).

In this step, we compute the Euclidean distance between each target and the optimum solution to measure proximity.

**Step 7**: Calculate the distance to negative-optimum Solution ( $\delta_{-}$ ).

Compute the Euclidean distance between each target and the negative-optimum solution to measure proximity.

**Step 8**: Determine the proximity to the Optimum Solution.

This step is to ascertain the degree of proximity between each assessment target and the optimum solution using the formula Eq. 10


(10) $$\mathrm{Proximity}=\frac{\delta_{-}}{\delta_{+}+\delta_{-}}$$

**Step 9**: Rank assessment targets.

Finally, with the calculated proximity value, targets can be ranked. The target with the highest relative closeness value is considered the most preferred or optimal choice.

### 3.6 Criteria determination

According to previous studies, over 50 indicators have been employed from different theoretical frameworks (2, 10, 11, 15, 18, 20). In general, some of the indicators have publicly available data that can be analyzed secondarily, while others must be subjectively evaluated by experts to obtain a particular score. However, an assessment performed using quantitative data may identify the one with the highest result statistically, but this strategy completely disregards individuals’ subjective judgment. To account for this issue, this study allows experts with different professional backgrounds to evaluate the importance of the evaluation indicators proposed in previous studies from different interest perspectives, and finally forms the evaluation indicators of this study to ensure that objective and subjective evaluation indicators have equal possibility of being included.

We first summarized the 50 evaluation indicators commonly used in previous studies (see Supplementary Material), and then recruited 30 professionals to rate the importance of each indicator. To ensure professionalism, purposive sampling was adopted. A brief introduction of the study was sent to a research institution specializing in urban governance studies at a university in Shanghai, China, inviting the director to assist us in recruiting 30 professional participants who would be willing to participate in an online survey. Out of the total sample size of 30 participants, 20 were female, and the remaining 10 were male. There are 10 individuals who have positions as faculty members specializing in public administration, 15 individuals who are engaged in research pertaining to disease prevention, and 5 individuals who serve as social workers within two distinct communities. The purposes of the study were introduced at the beginning of the questionnaire. Participants who responded to the survey after reading the introduction were considered to consent. A Likert-type scale from 1(not important at all) to 5 (very important) for each indicator was provided.

After calculating the average importance score for each indicator, the top 10 indicators were derived as the evaluation indicator system adopted in this study (see Table 2). The indicators obtained are characterized by the predominance of objective data, with supplementary descriptions of subjective evaluations. Of these, indicators *C1–C7* are based on objective data and *C8–C10* are based on individual subjective evaluation scores.

**Table 2:** Selected criteria for evaluating MPHPP.

Criteria		Definition
C1	Annual GDP	Macroeconomic foundations of the metropolis
C2	Annual budget of medical care	Amount of annual budgetary provision for public health care system of the metropolis
C3	Health care accessibility	Number of hospitals and health centers per capita
C4	Trained and professional personnel	Number of health care staff per capita
C5	Logistic volume	Freight volume per year
C6	Population density	Concentration of individuals within the metropolis
C7	Population mobility	Geographic movement of individuals
C8	Multisectoral coordination and communication	A functional multisectoral structure has been designed to facilitate the coordination and integration of pertinent sectors related to health security.
C9	Systematic analysis of surveillance data and prompt action	Functional surveillance mechanism for detecting, analyzing, and reporting data and action is implemented
C10	Non-pharmaceutical intervention (community-based)	Community-based health care education, intervention staff, capacities, procedures, and plans

## 4 A case study of metropolises evaluation with entropy-TOPSIS-IF

### 4.1 Target metropolises

**Figure 3:**
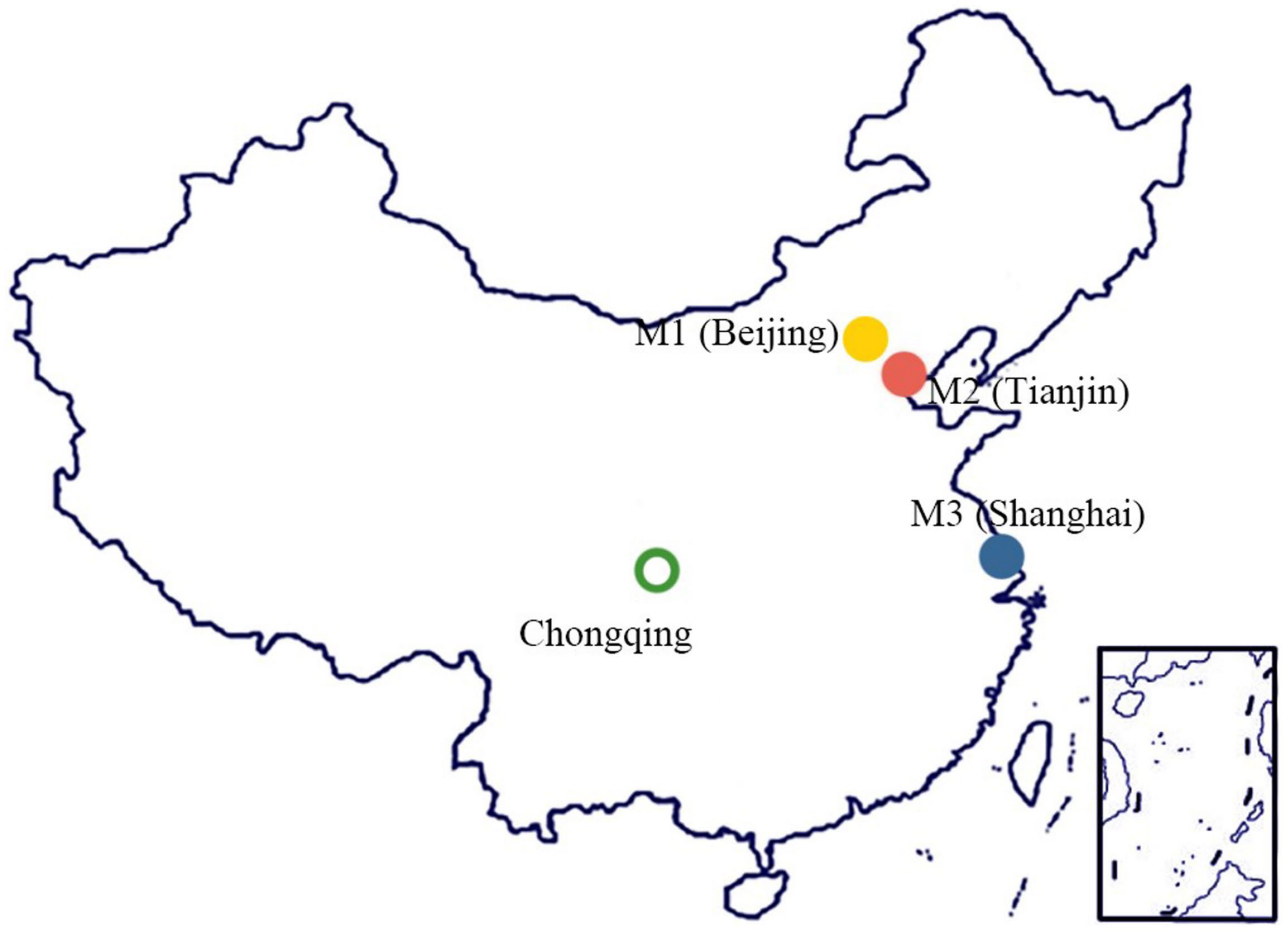
Location of the target metropolises.

To ascertain the legitimacy and effectiveness of the evaluation model proposed in this research, the preparedness of the four municipalities directly administered under the central government in China were initially assessed as potential evaluation targets. However, due to the geographical location of one of the municipalities in the country’s western region, there are notable disparities in its GDP per capita compared to the other three municipalities. To mitigate potential interference from varying levels of economic development, this study has deliberately excluded one municipality in the western region. Instead, as shown in Figure 3, the study evaluates and compares three metropolises in the eastern coastal area, which exhibit a relatively comparable level of economic development.

### 4.2 Expert selection and subjective criteria rating

Notably, public data of *C1–C7* are available on official metropolis statistics websites, while evidence-based data for *C8–C10* could not be obtained with the same method. Accordingly, two different approaches were utilized to evaluate three targets in the current study. First, for *C1–C7*, public data from city official websites were obtained and normalized. Second, purposive sampling was employed to choose experts who are experienced professionals from different sectors during the pandemic. Accordingly, one expert (see Table 3) from each target metropolis (*M1*, *M2*, and *M3*) was invited to complete an online questionnaire (from 1 = very weak to 5 = very well qualified) to evaluate the performances of the target metropolis with respect to *C8–C10*.

**Table 3:** Background information of three experts.

Surveyed	Job title	Current residence
Expert 1 (Ep1)	President of a public hospital	*M1*
Expert 2 (Ep2)	Department director of public health committee	*M2*
Expert 3 (Ep3)	Director of a community service office	*M3*

Expert 1 (*E1*) is a president of a public hospital from *M1*. Expert 2 (*E2*) is a department director of a public health committee, an administrative function of metropolitan public health system, from *M2*. To effectively illustrate the constructive impact of community engagement during the pandemic and its special function in the social environment within Mainland China, we invited a director of a community service office from *M3* as our expert 3 (*E3*).

### 4.3 Computation process and the results

Three experts (*E1*, *E2*, and *E3*) provided linguistic ratings to *C8–C10*, while scores of *C1–C7* were obtained from public official statistic data sets online (see Supplementary Material). Table 4 shows the results of linguistic ratings from experts. Equation (5) is utilized to compute the fuzzy weights for three metropolises. To illustrate, the aggregate rating for metropolis *M1* in relation to criteria *C8*, according to the evaluations provided by the three experts, is calculated as Eq. 11


(11) $$m_{ij}=\min_{k}\left(5,5,5\right),b_{ij}=\frac{1}{3}\overset{3}{\underset{k=1}{\sum }}\left(7,7,7\right),c_{ij}=\max_{k}\left(9,9,9\right)=\left(5,7,9\right)$$

**Table 4:** Experts’ subjective assessments of the three metropolises.

Criteria	Metropolis								
	*M1*			*M2*			*M3*		
	Ep1	Ep2	Ep3	Ep1	Ep2	Ep3	Ep1	Ep2	Ep3
C8	WQ	WQ	WQ	W	Q	Q	WQ	Q	VG
C9	Q	WQ	Q	W	Q	W	WQ	WQ	WQ
C10	Q	Q	WQ	WQ	Q	Q	Q	W	Q

Similarly, the collective evaluations for each of the three metropolises (*M1*, *M2*, *M3*) in relation to the three criteria are calculated. Table 5 provides the aggregate fuzzy decision matrix for the metropolis.

**Table 5:** The obtained metropolises’ fuzzy decision matrix.

Criteria	Metropolis		
	*M1*	*M2*	*M3*
C8	(5, 7, 9)	(1, 5, 7)	(3, 8.33, 9)
C9	(3, 7, 9)	(1, 5, 5)	(5, 7, 7)
C10	(3, 5, 7)	(5, 5, 7)	(1, 5, 9)

Next, data isotropy was performed. The TOPSIS method uses distance scales to measure sample gaps, which can lead to scale confusion if the data in one dimension are considered better when it is larger, and the data in the other dimension are considered better when it is smaller. Criteria *C5–C7* were turned into miniaturized indicators by the equation $x^{\prime }=\frac{1}{x}\;\left(x>0\right)$ . Then, all the data of each criterion were normalized by $p_{ij}=\frac{x_{ij}}{\sum_{i=1}^{n}x_{ij}}$ and have their weights calculated by entropy weight method. The weights calculated are shown in Table 6.

**Table 6:** Weight values of each criterion.

Criteria	C1	C2	C3	C4	C5	C6	C7	C8	C9	C10
Weight value	0.095	0.068	0.082	0.127	0.182	0.116	0.273	0.024	0.032	0.002

For the TOPSIS method, the best and the worst solutions were first identified using the equations Eqs. 12, 13


(12) $$\begin{array}{l} Z^{+}=\left(\max\left\{z_{11},z_{21},\cdots ,z_{n1}\right\},\max\left\{z_{12},z_{22},\cdots ,z_{n2}\right\},\cdots \max\left\{z_{1m},z_{2m},\cdots ,z_{\mathrm{nm}}\right\}\right)\\ =\left(Z_{1}^{+},Z_{2}^{+},\cdots ,Z_{m}^{+}\right) \end{array}$$

(13) $$\begin{array}{l} Z^{-}=\left(\min\left\{z_{11},z_{21},\cdots ,z_{n1}\right\},\min\left\{z_{12},z_{22},\cdots ,z_{n2}\right\},\cdots \min\left\{z_{1m},z_{2m},\cdots ,z_{\mathrm{nm}}\right\}\right)\\ =\left(Z_{1}^{-},Z_{2}^{-},\cdots ,Z_{m}^{-}\right) \end{array}$$

After that, the distance of each index toward the best ( $\delta_{i}^{+}$ ) and worst ( $\delta_{i}^{-}$ ) solutions are calculated by Eq. 14


(14) $$\delta_{i}^{+}=\sqrt{\overset{m}{\underset{j=1}{\sum }}w_{j}{\left(Z_{j}^{+}-z_{ij}\right)}^{2}},\;\delta_{i}^{-}=\sqrt{\overset{m}{\underset{j=1}{\sum }}w_{j}{\left(Z_{j}^{-}-z_{ij}\right)}^{2}}$$

( $w_{j}$ means the weight of the j_th_ index)

Finally, each metropolis’s data’s proximity to its best solution is calculated as follows, as shown in Table 7.


$$C_{i}=\frac{\delta_{i}^{-}}{\delta_{i}^{+}+\delta_{i}^{-}},0\leq C_{i}\leq 1.C_{i}\to 1\text{means the city has a better overall score. The metropolis was ranked depending on its}C_{i}\text{value.}$$

**Table 7:** Results of ranking scores of M1, M2, and M3.

	Positive ideal solution	Negative ideal solution	Score	Ranking
M1	0.368	0.446	0.548	1
M2	0.366	0.424	0.537	2
M3	0.533	0.199	0.272	3

**Figure 4:**
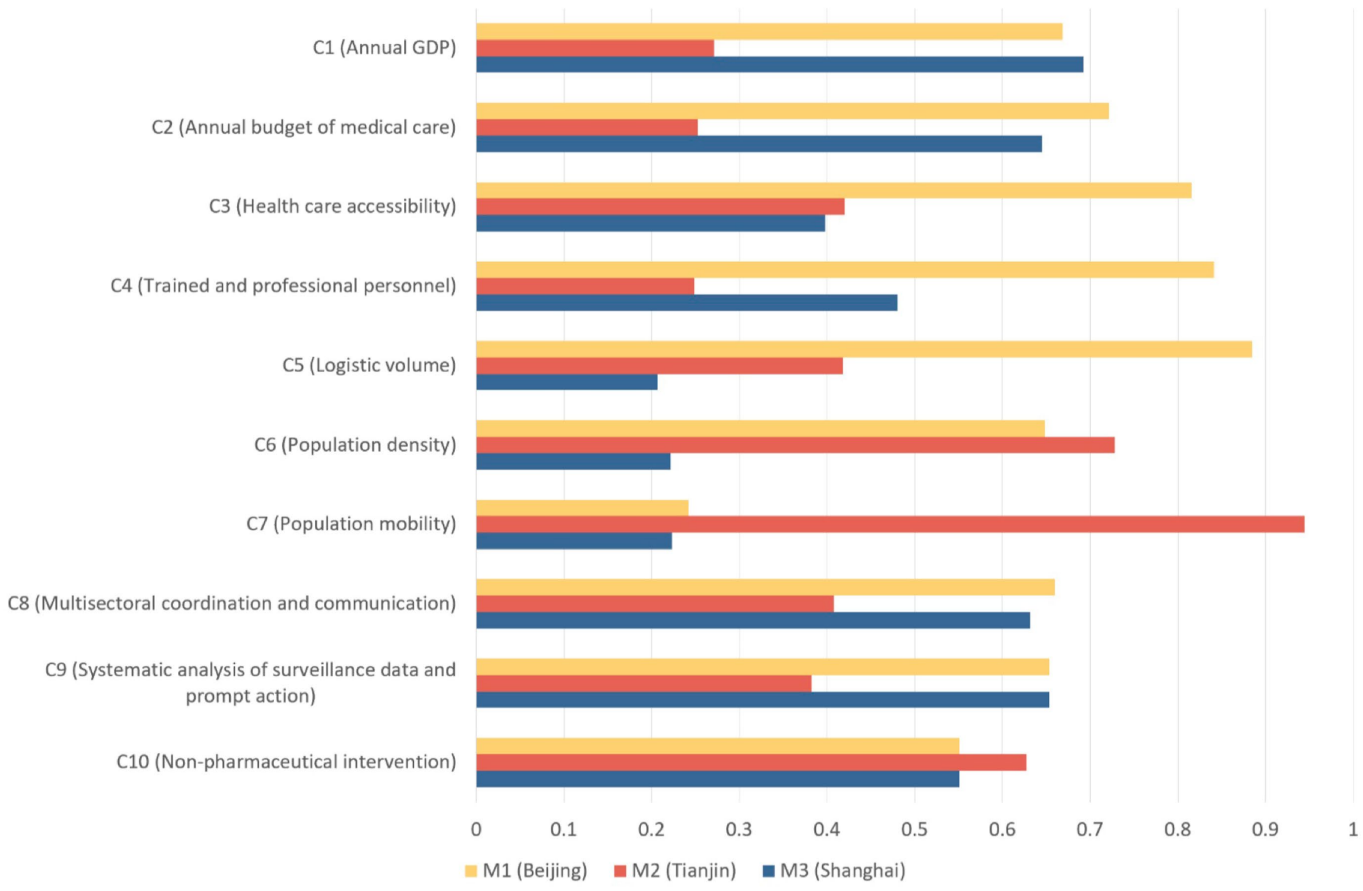
Detailed results of evaluation on *M1*, *M2* and *M3*.

As shown in Figure 4, *M1* achieved the best overall score, while *M3* underperformed on almost every criterion. Specifically, *M1* scored significantly higher in Logistic capacity and Health care accessibility, while *M3* had the worst performance in Population mobility and Population density.

It is worth noting that *M1* performed poorly together with *M3* in Population mobility. Despite scoring the lowest, *M3* had similar performance with *M1* in *C8*, *C9* and *C10*. This is probably because the two cities are among the most developed cities of China. This result is also in line with their scores on *C1* and *C2*.

Although *M3* had similar scores with *M1* and sometimes higher than *M2*, it had deplorable performance in *C5*, *C6* and *C7*, scoring a lot under both *M2* and *M3*. One noticeable finding is that *M2* scored unusual high scores on *C6* and *C7*, leading to an overall score only slightly lower than *M1*.

### 4.4 Sensitivity analysis

**Figure 5:**
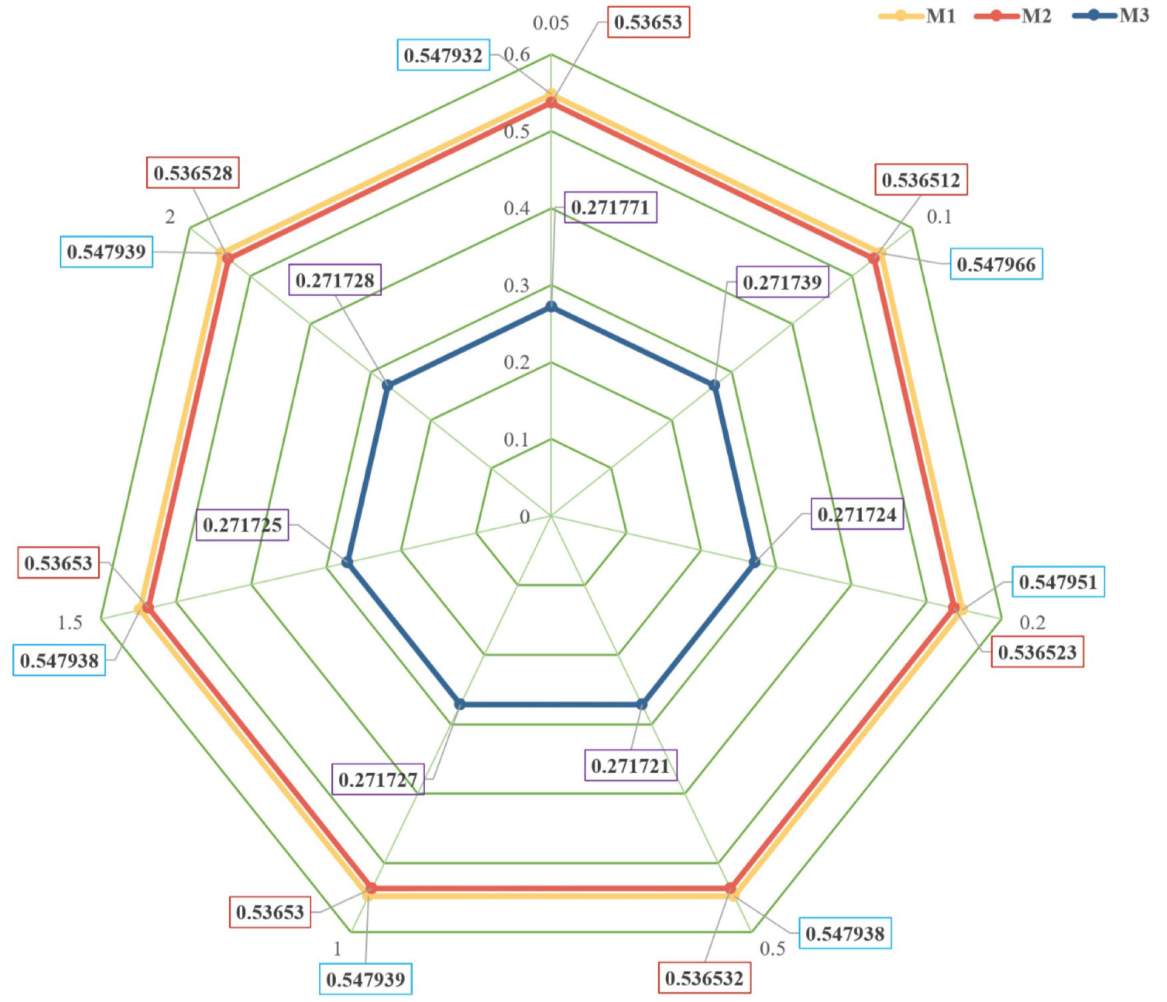
Sensitivity analysis results.

The primary objective of sensitivity analysis is to scrutinize the extent to which minor modifications in the individual weights assigned throughout the pair-wise comparison procedure influence the overall judgment. The inquiry at hand can be resolved by making modest adjustments to the weights’ values and thereafter evaluating the resultant impact on the conclusion. This is advantageous in scenarios where ambiguities arise in delineating the significance of certain aspects. To assess the sensitivity of each criterion in the entropy-TOPSIS-IF for MPHPP, we adjust the unitary ratio of each criterion weight. Alterations for the weights of criteria *C1* to *C10* were done depending on the unitary ratios $\beta_{1}$ . $\beta_{1}$ ranged from 0.05 to 2, as presented in Table 8. The unitary ratio is calculated by $\frac{W_{k}^{\prime }}{w_{k}}$ . The scores of each criterion after weight adjustment for each city are shown in Table 8. Little differences were seen after the adjustment. In addition, as shown in Figure 5, the trend was fluent, indicating that the model is stable and insensitive to changes.

**Table 8:** Results of sensitivity analysis.

	Unitary ratio						
	0.05	0.1	0.2	0.5	1	1.5	2
*C1*	0.005	0.009	0.019	0.047	0.095	0.142	0.189
*C2*	0.003	0.007	0.014	0.034	0.068	0.101	0.135
*C3*	0.004	0.008	0.016	0.041	0.082	0.123	0.164
*C4*	0.006	0.013	0.025	0.064	0.127	0.191	0.254
*C5*	0.009	0.018	0.036	0.091	0.182	0.273	0.364
*C6*	0.006	0.012	0.023	0.058	0.116	0.174	0.232
*C7*	0.014	0.027	0.055	0.136	0.273	0.409	0.545
*C8*	0.001	0.002	0.005	0.012	0.024	0.036	0.048
*C9*	0.002	0.003	0.006	0.016	0.032	0.048	0.064
*C10*	0	0	0	0.001	0.002	0.003	0.005
*M1*	0.548	0.548	0.548	0.548	0.548	0.548	0.548
*M2*	0.537	0.537	0.537	0.537	0.537	0.537	0.537
*M3*	0.272	0.272	0.272	0.272	0.272	0.272	0.272
Ranking	1 > 2 > 3	1 > 2 > 3	1 > 2 > 3	1 > 2 > 3	1 > 2 > 3	1 > 2 > 3	1 > 2 > 3

## 5 Discussion

### 5.1 The novel entropy-TOPSIS-IF model

The main purpose of the current study was to propose an innovative, math modeling-based and comprehensive framework to evaluate MPHPP integrating SBA and DBA approaches. This evaluation framework offers practical solutions for the comprehensive evaluation of a hybrid evaluation system covering both linguistic and quantitative indicators.

According to the validation of the case study, entropy-TOPSIS-IF model demonstrates the following advantages: (1) data-based quantitative and expert-evaluated linguistic indicators are incorporated in entropy-TOPSIS-IF to build a more comprehensive and versatile evaluation framework; (2) lie in its integration of linguistic ratings for criteria *C8–C10*, quantified via the aggregation of fuzzy decisions, with public official statistical data for measures *C1–C7*, ensuring the comprehensiveness of entropy-TOPSIS-IF in assessing MPHPP; (3) flexibility is also a noteworthy advantage of entropy-TOPSIS-IF, it can be universally applied in various situations without considering expert decision-making or the availability of quantitative data; (4) the findings of the sensitivity analysis indicated that slight variations in the weights of criteria *C1–C10* had minimal impact on evaluation outcomes. This observation provides additional evidence supporting the high robustness and stability of entropy-TOPSIS-IF.

### 5.2 Limitation and policy recommendations

Meanwhile, we also noted the limitations of this study. First, fuzzy set theory highly relies on the membership functions developed by experts based on their experience to describe decision-making. This approach introduces a degree of subjectivity and may inevitably result in the loss of some semantic information. Investigating alternative approaches such as deep learning based (37) or large language model-based ones (38) for quantifying linguistic data more efficiently and unbiasedly might be a promising avenue for future study.

In addition, we only recruited 3 experts to rate the *C8–C10* indicators in the case study, which can lead to unconvincing results. Future studies need to include as many stakeholders as possible in the evaluation. The application of mixed research designs in evaluation studies can also be considered. The use of qualitative research methods, such as focus group interviews, to supplement the description or to validate the results derived from quantitative evaluations can also be utilized to increase the robustness of the evaluation study in terms of triangulating evidence across the board.

Inconsistent with our previous perception, results of our case study indicated that *M3* is the worst performing metropolis. The worst overall score is majorly due to low score on the most heavily weighted indicator population mobility. Its scores were also poor on other indicators with high weights, such as medical professionals and logistics capacity. The main reason for this result is that this study used the entropy to calculate weight of each criterion, and the three subjective evaluation indicators are not well differentiated, as the entropy weighting method tends to favor factors that have large discrepancy in evaluation targets, the three subjective evaluation indicators *C8–C10* were assigned lower weights; while the objective data indicators *C1–C7*, which are in the majority, are well differentiated and assigned higher weights. Being the China’s largest economy metropolis, *M3* fails to gain advantage on several more highly weighted indicators. Nevertheless, in addition to being ranked first in terms of GDP, *M3* scores well on the subjective evaluation indicators (*C8–C10*). This result suggests that *M3* must figure out how to counteract the risks posed by its huge logistics and people flow. Accordingly, this result provides strong evidence for future policymaking to address similar public health risks. Therefore, three policy suggestions should be considered based on the current study. First, the joint meeting and multi-party cooperation mechanism of different government sectors need to be developed and implemented regularly and continuously. This system can ensure that the utilization of resources, such as medical care, can be maximized, and it can also avoid the problem that various departments focusing only on local aspects and neglecting overall situation. Second, strengthen the data driven monitoring on early warning signs, and decision making with systematic analysis. The simulation of large-scale public risks occurrence can provide critical preparation time for response. In the meanwhile, public can gain psychological preparation to prevent triggering panic from sudden risks. Third, lockdown is effective on reducing pandemic impact, but it is clearly detrimental to the urban economy and human health, both physically and mentally. Non-medical interventions are particularly important for the older adult and children, who are the most vulnerable population in the city. Communities should be prepared for non-medical interventions and family function support in terms of trained or professional personnel and resource allocation.

## 6 Conclusion

This article introduces a novel evaluation framework entropy-TOPSIS-IF for conducting a multicriteria decision-making analysis to assess the level of preparation of metropolitan areas in the context of public health emergencies, specifically focusing on pandemics threat. The contribution of entropy-TOPSIS-IF lies in its practical applicability to provide an evaluation approach under both partial and complete lack of subjective/objective information. We applied it in a case study evaluating the MPHPP of three China metropolises to show its effectiveness. Results illustrated that the fusion of the two types of indicators can reveal problems that traditional evaluation cannot, and the sensitivity analysis results further confirmed the feasibility of this new method.

## Data availability statement

The original contributions presented in the study are included in the article/Supplementary materials, further inquiries can be directed to the corresponding author.

## Author contributions

JL: Data curation, Writing – original draft, Writing – review & editing, Investigation, Resources, Software, Visualization. AL: Data curation, Formal analysis, Investigation, Software, Writing – initial draft, Writing – review& editing. XL: Writing – original draft, Conceptualization, Supervision, Writing – review & editing. HL: Visualization, Writing – review & editing. WL: Writing – review & editing, Conceptualization, Project administration, Visualization. WC: Conceptualization, Writing – review & editing.
