# Supplementary material for: Evaluating the metropolitan public health preparedness for pandemics using entropy-TOPSIS-IF
Source: Front Public Health. 2024 Mar 7;12:1339611. doi: 10.3389/fpubh.2024.1339611 (PMC10955133; doi:10.3389/fpubh.2024.1339611)
Supplement: Supplementary file 1 [file Data_Sheet_1.pdf]

Supplementary Material

1 Supplementary Data

1.1 Supplementary Tables

Supplementary Table 1. Indicators of MPHPP Assessment for experts to conduct importance rating (*Notes: all indicators were collected from previous relevant studies.*)

**Categories**	**Dimensions**	**Indicators (*N* = 50)**
Metropolitan statistics	GDP and budgeting	Annual GDP
		Annual budget of medical care
	Logistic volume	Freight volume per year
	Population mobility	Geographic movement of individuals
Prevent public health emergencies	Legislations, policies, administrative frameworks	Legislation, laws, regulations, policy, administrative requirements are available in all relevant health security sectors
		The municipality has assessed, adjusted and aligned its legislation, laws, regulations, policy, and administrative requirements in all relevant health security sectors.
	Financing and resources	Financing is available for the development and implementation of health security capacities in a municipality.
		Financing mechanisms and funds are available for the timely response to public health emergencies in a municipality.
	Multisectoral communications	A functional multisectoral mechanism is established for the coordination and integration of relevant health security sectors.
	Multihazard risk assessment	Multihazard risk assessment to determine the nature and extent of health security risks for the municipality.
		Health security risk information is disseminated to relevant sectors and applied to administrative decisions.

	Immunization	Municipality has the capacity to access, store and deliver vaccines to the entire population of the municipality.
		Surveillance systems for immunization coverage are established and functional.
Detect public health emergencies	Laboratory systems	Laboratory testing for the detection of priority diseases is established and implemented through local laboratories or established laboratory networks.
		Laboratory specimen referral and transport systems are established and functional.
		Laboratory quality system is established and implemented.
	Surveillance systems	Functional surveillance systems for identifying potential events of concern for public health and health security are established and implemented.
		Surveillance systems are supported by the use of electronic tools.
		Surveillance systems for the detection and monitoring of foodborne diseases and food contamination are established and implemented.
		Surveillance systems for zoonotic diseases and pathogens are established and implemented.
		Systematic analysis of surveillance data for action is implemented.
	Reporting protocols, systems and networks	Municipal reporting protocols and systems.
	Human resources for health security	A current multisectoral workforce strategy is in place.
		Human resources are available to effectively implement health security activities.
		In-service training requirements.

Respond to public health emergencies	Municipal emergency preparedness	Emergency response resources are identified and mapped.<br><br>Multisectoral multihazard emergency preparedness plans and measures are developed and implemented.
	Municipal emergency response and incident management system	Emergency response coordination mechanisms.<br><br>Emergency operations center (EOC) capacities, procedures, and plans.
	Non-pharmaceutical interventions	Personal non-pharmaceutical intervention capacities, procedures, and plans.<br><br>Community non-pharmaceutical intervention capacities, procedures and plans.<br><br>Environmental non-pharmaceutical intervention capacities, procedures and plans.
	Health care delivery	Case management procedures implemented for health security hazards.<br><br>Medical surge capacity is available for the timely response to public health emergencies.
	Medical countermeasures and health personnel deployment	System in place for activating and coordinating medical countermeasures during a public health emergency.<br><br>System in place for activating and coordinating health personnel during a public health emergency.
	Risk communication	Risk communication systems for public health events and emergencies.<br><br>Internal and partner coordination for emergency risk communication.<br><br>Communication engagement with public and media during emergencies.<br><br>Communication engagement with affected communities during emergencies.

		Addressing perceptions, risky behaviors, and misinformation.
	Human resource management	Systems are in place for managing and coordinating public health and security authorities.
		Systems are in place for managing and coordinating volunteers during a public health emergency.
	Recovery and rehabilitation	A multisectoral recovery plan is established and functional to guide recovery operations and efforts following an event or emergency.
Social policy resilience	Population density	Concentration of individuals within the metropolis on the pandemic
	unemployment	Number of unemployed in urban areas
	Public administration practitioners	Number of policy officers under the pandemic
	Medical insurance rate	Number of persons covered by basic medical insurance
	Health care accessibility	Number of hospitals and health centers per capita
	Trained and professional personnel	Number of health care staff per capita

1.2 Data source for the three target metropolises M1, M2 and M3 .

M1: https://nj.tjj.beijing.gov.cn/nj/main/2021-tjnj/zk/indexch.htm

M2: https://stats.tj.gov.cn/tjsj_52032/tjnj/

M3: https://tjj.sh.gov.cn/tjnj/
